# Diffusion-Weighted Imaging and Mapping of T1 and T2 Relaxation Time for Evaluation of Chronic Renal Allograft Rejection in a Translational Mouse Model

**DOI:** 10.3390/jcm10194318

**Published:** 2021-09-23

**Authors:** Martina Schmidbauer, Song Rong, Marcel Gutberlet, Rongjun Chen, Jan Hinrich Bräsen, Dagmar Hartung, Martin Meier, Frank Wacker, Hermann Haller, Faikah Gueler, Robert Greite, Katja Derlin

**Affiliations:** 1 Institute for Diagnostic and Interventional Radiology, Hannover Medical School, Carl-Neuberg-Straße 1, 30625 Hannover, Germany; gutberlet.marcel@mh-hannover.de (M.G.); hartung.dagmar@mh-hannover.de (D.H.); wacker.frank@mh-hannover.de (F.W.); Derlin.Katja@mh-hannover.de (K.D.); 2 Clinic for Nephrology, Hannover Medical School, Carl-Neuberg-Straße 1, 30625 Hannover, Germany; rong.song@mh-hannover.de (S.R.); chen.Rongjun@mh-hannover.de (R.C.); haller.hermann@mh-hannover.de (H.H.); Gueler.Faikah@mh-hannover.de (F.G.); Greite.Robert@mh-hannover.de (R.G.); 3 Institute for Pathology, Hannover Medical School, Carl-Neuberg-Straße 1, 30625 Hannover, Germany; braesen.jan@mh-hannover.de; 4 Institute for Laboratory Animal Science and Central Animal Facility, Central Animal Facility, Hannover Medical School, Carl-Neuberg-Straße 1, 30625 Hannover, Germany; meier.martin@mh-hannover.de

**Keywords:** magnetic resonance imaging (MRI), T2 mapping, T1 mapping, diffusion-weighted imaging, apparent diffusion coefficient, kidney transplantation, rejection, inflammation, fibrosis

## Abstract

We hypothesized that multiparametric MRI is able to non-invasively assess, characterize and monitor renal allograft pathology in a translational mouse model of chronic allograft rejection. Chronic rejection was induced by allogenic kidney transplantation (ktx) of BALB/c-kidneys into C57BL/6-mice (*n* = 23). Animals after isogenic ktx (*n* = 18) and non-transplanted healthy animals (*n* = 22) served as controls. MRI sequences (7T) were acquired 3 and 6 weeks after ktx and quantitative T1, T2 and apparent diffusion coefficient (ADC) maps were calculated. In addition, in a subset of animals, histological changes after ktx were evaluated. Chronic rejection was associated with a significant prolongation of T1 time compared to isogenic ktx 3 (1965 ± 53 vs. 1457 ± 52 ms, *p* < 0.001) and 6 weeks after surgery (1899 ± 79 vs. 1393 ± 51 ms, *p* < 0.001). While mean T2 times and ADC were not significantly different between allogenic and isogenic kidney grafts, histogram-based analysis of ADC revealed significantly increased tissue heterogeneity in allografts at both time points (standard derivation/entropy/interquartile range, *p* < 0.05). Correspondingly, histological analysis showed severe inflammation, graft fibrosis and tissue heterogeneity in allogenic but not in isogenic kidney grafts. In conclusion, renal diffusion weighted imaging and mapping of T2 and T1 relaxation times enable detection of chronic renal allograft rejection in mice. The combined quantitative assessment of mean values and histograms provides non-invasive information of chronic changes in renal grafts and allows longitudinal monitoring.

## 1. Introduction

Chronic graft rejection is one of the major causes of long-term graft loss after kidney transplantation (ktx) and is still poorly understood [1,2,3]. The clinical diagnosis is usually suggested by slowly progressive decline of graft function, as manifested by rising plasma creatinine concentration, increasing proteinuria and worsening hypertension. However, the reliance on these clinical features commonly results in the late identification of chronic renal allograft nephropathy, frequently culminating in allograft loss [4].

For diagnosis of renal pathology kidney biopsy is often required as the reference standard. In the complex pathogenesis of chronic allograft damage antigen-dependent and –independent factors initiate inflammation and fibrosis of the kidney graft, classified according to the Banff criteria [5]. However, this procedure is prone to sampling errors, which can provide false negative results [6]. Furthermore, repeated biopsies are difficult to implement due to their invasiveness and poor patient acceptance.

Magnetic resonance imaging (MRI) is a promising technique to assess and monitor kidney allograft function without invasiveness [7,8]. In addition to morphological features, functional and microstructural information of the renal allograft, including oxygenation, tissue edema, glomerular filtration, cellularity and perfusion, can be provided [9]. Furthermore, MRI is independent from the operator, can be obtained without administration of contrast agent and without radiation exposure, so that serial measurements can be performed.

In the past, T1 and T2 mapping as well as diffusion-weighted imaging (DWI) have been investigated to evaluate renal function and morphology in different types of kidney disease [10,11,12,13,14]. While conventional MRI only enables a qualitative image interpretation based on signal intensity analysis, in mapping technique a voxel-wise evaluation allows direct quantification of tissue composition. Quantitative relaxation values are illustrated in greyscale- or color-coded parameter maps and can be directly compared across subjects and examinations under the same condition. T1 and T2 times are dependent on the molecular environment and total amount of water molecules within a given tissue. Previous clinical studies have shown that T1 mapping could be helpful for identifying acute kidney injury and for detection of chronic kidney disease in mice [15,16,17]. Additionally, recent human clinical studies have shown promising results of renal T1 mapping for the detection of fibrosis and assessment of graft function after kidney transplantation [18,19,20]. In animal models of acute kidney injury following ischemia reperfusion, injury elevated T2 times were associated with inflammation and tissue edema [21,22,23]. DWI depends on Brownian motion of water protons and is quantified by the apparent diffusion coefficient (ADC). DWI is promising to detect renal allografts with impaired function based on lower ADC values [13,24]. In addition, DWI holds potential for the assessment of chronic tubulointerstitial fibrosis associated with chronic kidney disease [18,25,26].

Until now, chronic graft rejection has rarely been studied in animal models. We adopted a translational mouse model of chronic rejection after ktx, which was initially described by Zarjou et al. [27]. This model shows features of both antibody mediated- (ABMR) and T-cell-mediated rejection (TCMR) with severe peritubular capillaritis and chronic tubulointerstitial fibrosis as well as severe renal perfusion impairment as detected by fMRI in allografts 6 weeks after ktx [28].

We hypothesized that multiparametric MRI can be used to assess and monitor chronic renal allograft rejection. Therefore, we longitudinally evaluated quantitative T1-, T2-mapping and DWI as potential imaging marker for chronic renal allograft pathology.

## 2. Materials and Methods

### 2.1. Animals

C57BL/6JHan-ztm (genotype: H2b) male mice were used as recipients, BALB/cJ (genotype: H2d) male mice served as kidney donors. All mice were 11–16 weeks of age. Mice were bred in the Institute of Laboratory Animal Sciences Hannover Medical School, were kept under standardized conditions of 14/10-h day and night cycle and had free access to food and drinking water. All experiments were approved by the animal protection committee of the Lower Saxony state department for food safety and animal welfare (LAVES) (ethical approval: 33.12-42502-04-14/1569).

### 2.2. Kidney Transplantation in Mice

Ktx was conducted by an experienced microsurgeon under isoflurane (3–5% induction and 1.5% maintenance) inhalation anesthesia and butorphanol (1.5 mg s.c.) for analgetic treatment as described previously [29]. Briefly, after midline abdominal incision the left kidney of the donor, a cuff of the infrarenal aorta, the renal vein and the ureter were removed en bloc and transplanted into the left lower abdomen of the recipient after the left native kidney of the recipient had been removed. Both aortic and venous end-to-side anastomosis were performed and the ureter was implanted into the bladder. The right native kidney of the recipient remained in situ to maintain renal function during the observation period. After surgery, mice were monitored daily. Reasons for premature study termination were reduced activity or food intake and hind limb paralysis. In addition, animals with severe postoperative hydronephrosis due to reflux or obstruction, as well as complete graft infarction, were excluded.

Chronic renal allograft rejection was induced by transplantation of BALB/c-donor kidneys into C57BL76-recipients (*n* = 23). For isogenic transplantation, C57BL76 mice served as donor and recipients (*n* = 18). Cold and warm ischemia times were 30 min, respectively, in both groups. Pathophysiological changes were investigated by MRI at week 3 and 6 after surgery. In addition, kidneys of BALB/c mice without surgery, which served as kidney donors in the allogenic group, were examined as healthy control (*n* = 22).

Histological changes after allogenic and isogenic ktx were evaluated after the observation period of 6 weeks (i.e., one day after the last MRI) in a subset of animals (*n* = 8 with allogenic and *n* = 6 with isogenic ktx).

### 2.3. Functional MRI for Assessment of Renal Allograft Pathophysiology

For characterization of renal allograft pathology all mice were examined by MRI using a 7 Tesla small-animal MRI scanner (Bruker; Pharmascan, PHS70/16, Ettlingen, Germany) with a circularly polarized volume coil (Bruker T10327 V3) or a 4-element phased-array surface receive coil (Bruker T20027 V3). Anesthesia was induced with 3% and maintained with 1–2% isoflurane during MRI. Respiration was monitored and kept between 50–60 breaths per minute. For measurement of T1 relaxation time of renal tissue, a fat-saturated single-shot inversion recovery sequence with multiple inversion times, a non-selective inversion pulse and an echoplanar (EPI) readout was used: TR/TE = 18,000/16.4 ms, 13 inversion times (30, 100, 200, 300, 500, 700, 1000, 1200, 1500, 2000, 3000, 5000, 8000 ms), matrix = 128 × 128, field of view (FOV) = 35 × 35 mm^2^, slice thickness = 2 mm, number of slices = 1. For T2 mapping, a fat-saturated, respiratory-triggered multi-echo turbo spin-echo sequence was acquired: nominal TR = 1000 ms, effective TR = 2000–3000 ms (depending on respiration rate), 7 TE = 11–77 ms, matrix = 256 × 256, FOV = 35 × 35 mm^2^, slice thickness = 2 mm. DWI was acquired using a respiratory-triggered, fat-saturated EPI sequence with the following parameters: nominal TR = 800 ms, effective TR/TE = 2000–3000/22–35 ms (depending on respiration rate and coil), 7 b-values = 0–800 s/mm^2^, matrix = 128 × 128 − 172 × 172, FOV = 35 × 35 mm^2^, slice thickness = 1.2–2 mm. All images were acquired in an oblique coronal slice, oriented along the long axis of the kidney.

Parameter maps of T1 and T2 relaxation time and the ADC (mono-exponential fit) were calculated with MATLAB software (MathWorks, Natick, MA, USA) as described previously (21; 15). Regions of interest (ROIs) were placed manually in the renal cortex, the outer stripe (OSOM) and the inner stripe of the outer medulla (ISOM) of T1 and T2 maps using Osirix software (v.6.0.2, Pixmeo, Bernex, Switzerland). As anatomic differentiation between the ISOM and OSOM in ADC maps was not possible, ROIs were placed in the entire outer medulla (OM). Mean values for each region as well as the cortico-medullary difference (CMD, difference between ISOM and cortex) were calculated. For additional histogram analysis, ROIs covering the entire kidney were placed on ADC maps. Probability density of the ADC was estimated using a kernel method (‘ksdensity’ function) of MATLAB (v9.2, Mathworks, Natick, MA, USA). The following parameters were derived from the histograms: (1) mean (average value of the pixels within the ROI); (2) standard deviation (width of the histogram or degree of variation/dispersion from the average); (3) kurtosis, which is the degree of the tailedness of a distribution; (4) skewness, which is a measure of asymmetry of a distribution; and (5) entropy, which is a textural-based measure of irregularity.

### 2.4. Histology and Immunohistochemistry for Assessment of Renal Allograft Morphology

Paraffin embedded renal tissue were cut into 2 µm sections and stained with Masson Trichrome (MTC) revealing renal fibrosis in blue. CD3 (hamster anti mouse CD3, eBioscience) was used to detect T-lymphocytes and fibronectin (rabbit anti mouse, abcam) to investigate fibrosis. Primary antibody incubation was 1 h at room temperature followed by the secondary antibody (Alexa Fluor^®^ 594; Thermo Fisher Scientific, Waltham, MA, USA) for another hour. Slides were mounted with DIANOVA mounting medium containing DAPI for nuclear staining. CD3 positive T-lymphocytes and interstitial fibrosis were quantified semi-quantitatively with a score from 0 to 4 corresponding to mild (1), moderate (2), severe (3) and very severe (4) inflammatory cell infiltration or interstitial fibrosis.

### 2.5. Statistical Analysis

Statistical analysis was performed with GraphPad Prism 9.0 (GraphPad Software, Inc., La Jolla, CA, USA) and SPSS version 22 (IBM Corporation, Armonk, NY, USA). Normal distribution of MRI values was confirmed by the Shapiro–Wilk test. Differences between groups were assessed by using one-way ANOVA and Tukey’s post hoc test or unpaired *t*-tests where appropriate. Changes between timepoints within one group were analyzed with the paired *t*-test. All values are given as mean ± standard error of the mean (SEM). *p*-values < 0.05 were accepted as significant.

## 3. Results

### 3.1. Characterization of Tissue Composition in Kidney Grafts by Mapping of T1 and T2 Relaxation Times with fMRI

#### 3.1.1. T1-Mapping

**Figure 1:**
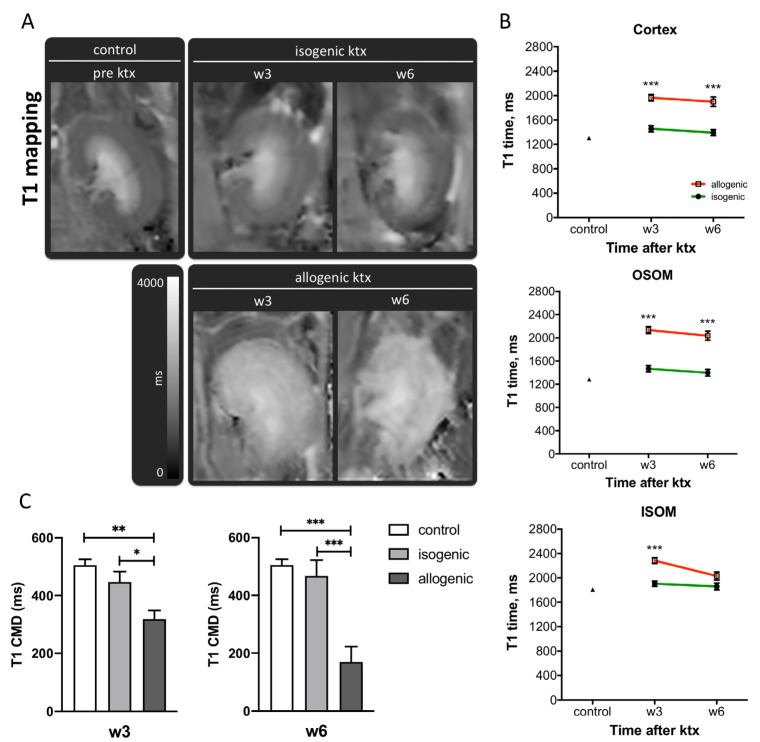
T1 relaxation time of renal tissue after ktx. Representative T1 maps of a normal control kidney, isogenic and allogenic kidney grafts at week 3 (w3) and week 6 (w6) after transplantation are shown (**A**). Image size, window level and width are similar. Means ± SEM of T1 values in the cortex, the outer stripe of the outer medulla (OSOM) and the inner stripe of the outer medulla (ISOM) (**B**) as well as the cortico-medullary difference (CMD) (**C**) are shown. Significant differences between isogenic and allogenic ktx are indicated: * *p* < 0.05, ** *p* < 0.01, *** *p* < 0.001. Control *n* = 22, isogenic ktx *n* = 7, allogenic ktx *n* = 5.

In the normal kidney, T1 relaxation times were low in cortex (1304 ± 10 ms) and OSOM (1283 ± 11 ms) and high in the ISOM (1808 ± 19 ms). Three and six weeks after isogenic ktx, T1 relaxation times were slightly but not significantly elevated when compared to controls in all anatomical layers (week 3: *p* = 0.09, week 6: *p* = 0.48). Allogenic ktx was associated with severe elevation of T1 relaxation time in all anatomical layers when compared to control kidneys (*p* < 0.01) as well as to isogenic kidney grafts (*p* < 0.001) at both time points (Figure 1A,B and Table 1). In the OSOM for example, T1 values in allografts after 3 weeks were 2134 ± 61 ms compared to 1466 ± 56 ms in isografts and 1283 ± 11 ms in control kidneys. In allografts but not in isografts, the physiological CMD was significantly reduced (Figure 1C).

#### 3.1.2. T2-Mapping

**Figure 2:**
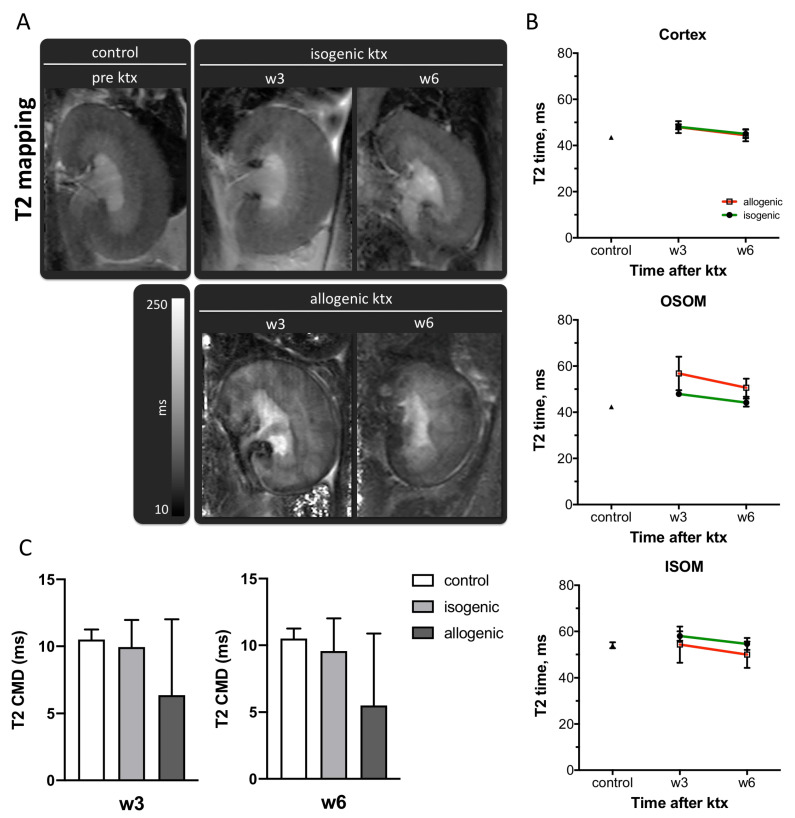
T2 relaxation time of renal tissue after ktx. Representative T2 maps of a normal control kidney, isogenic and allogenic kidney grafts at week 3 (w3) and week 6 (w6) after transplantation are shown (**A**). Image size, window level and width are similar. Means ± SEM of T2 values in the cortex, the outer stripe of the outer medulla (OSOM) and the inner stripe of the outer medulla (ISOM) (**B**) as well as the cortico-medullary difference (**C**) are indicated. Control *n* = 22, isogenic *n* = 5, allogenic *n* = 8.

T2-relaxation times in normal kidneys were 44 ± 1 ms in the renal cortex, 42 ± 1 ms in the OSOM and 54 ± 1 ms in the ISOM. The CMD was 11 ± 1 ms (Figure 2A,B and Table 1). Allogenic ktx was associated with an elevation of T2 relaxation time in the OSOM after three weeks (control: 42 ± 1 ms, isogenic ktx: 48 ± 1 ms, allogenic ktx: 57 ± 7 ms) and six weeks (control: 42 ± 1 ms, isogenic ktx: 44 ± 2 ms, allogenic ktx: 51 ± 4 ms). Statistical significance was only reached between allogenic grafts three weeks after surgery and controls (*p* < 0.001). Due to tissue heterogeneity in allogenic grafts, the CMD had a wide range at both timepoints (week 3: −12.2 ms to 17.7 ms, week 6: −8.3 ms to 17.1 ms). In isogenic grafts the CMD was preserved at both time points (Figure 2C).

### 3.2. Assessment of Inflammation and Heterogeneity of Graft Pathology by DWI

#### 3.2.1. Renal Diffusivity

**Figure 3:**
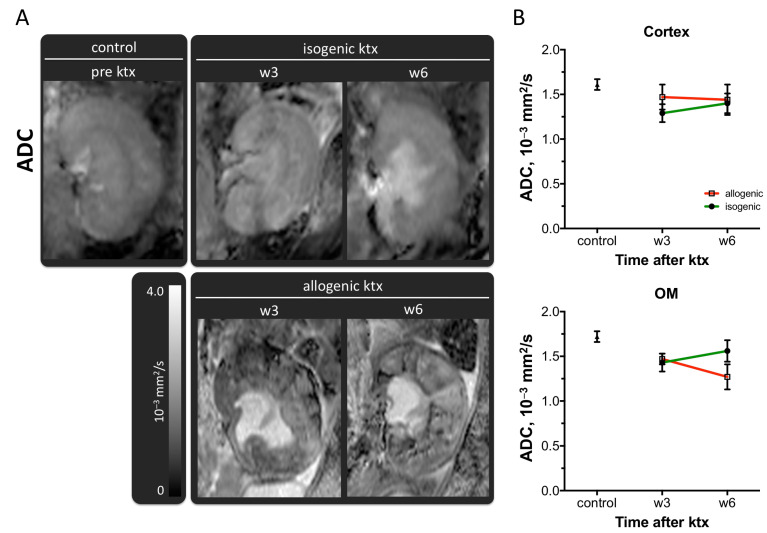
Apparent diffusion coefficient (ADC) of renal tissue after ktx. Representative ADC maps of a normal control kidney, isogenic and allogenic kidney grafts at week 3 (w3) and week 6 (w6) after transplantation are shown (**A**). Image size, window level and width are similar. Means ± SEM of ADC-values in the cortex and the outer medulla (OM) are given (**B**). Note the obvious heterogeneity of the renal tissue in the ADC map after allogenic ktx compared to the homogeneously parenchyma in the isografts.

Mean cortical ADC of isografts and allografts at week 3 and 6 after transplantation were not significantly different (Figure 3B and Table 1). The ADC of the OM was significantly decreased in allografts 6 weeks after ktx compared to control (1.27 ± 0.14 10^–3^ mm^2^/s vs. 1.72 ± 0.06 10^–3^ mm^2^/s, *p* < 0.01). However, medullary ADC in isografts without rejection and allografts with rejection was not significantly different.

#### 3.2.2. Assessment of Tissue Heterogeneity by Histogram Analysis of ADC Maps

Renal appearance of allogenic kidney grafts was obviously heterogeneous and clearly different from isogenic kidney grafts and control kidneys (Figure 3A). ADC maps of allogenic kidney grafts showed areas of high and low ADC values side-by-side in the cortex and the OM and destruction of renal anatomy.

**Figure 4:**
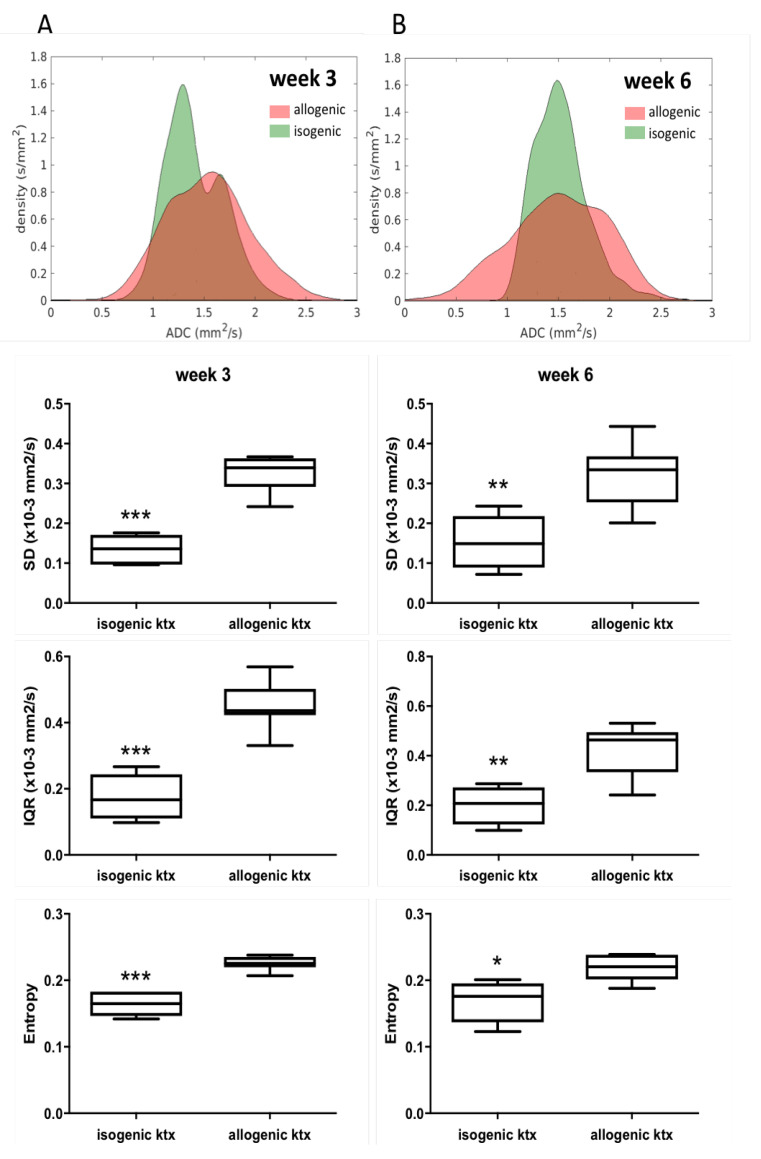
Assessment of tissue heterogeneity by histogram analysis of ADC maps. Cumulative histograms in allografts and isografts at week 3 (**A**, left column) and 6 (**B**, right column) after ktx show increased tissue heterogeneity after allogenic ktx. Box plots show parameters of heterogeneity (SD, IQR and entropy) derived from the quantitative histogram. Each box stretches from the 25th percentile at lower edge to the 75th percentile at upper edge; the median is shown as a line across. Significant differences are indicated with * *p* < 0.05, ** *p* < 0.01 and *** *p* < 0.001.

In order to quantify tissue heterogeneity and to verify the visual MRI findings, histogram-based analysis of renal ADC maps was performed. Histograms showed a wider distribution of ADC values in allografts compared to isografts (Figure 4). At week 3 and 6, quantitative parameters of heterogeneity derived from histogram analysis such as standard derivation (SD), interquartile range (IQR) and entropy were significantly increased in allografts with chronic rejection compared to isografts (week 3: *p* < 0.001 each; week 6: *p* < 0.05–0.01). Kurtosis and skewness were not significantly different between groups (Table 2).

### 3.3. Histology and Immunohistochemistry in Kidney Grafts

**Figure 5:**
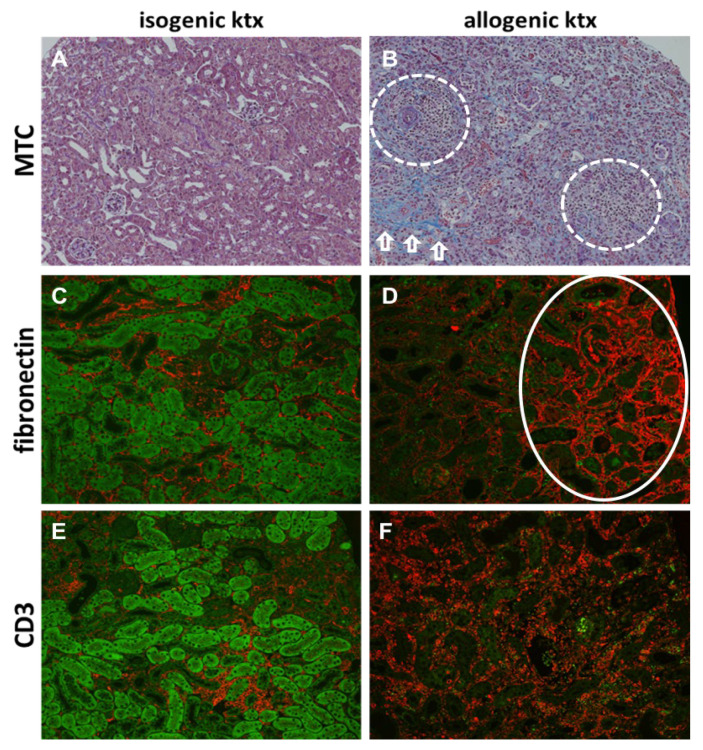
Histological changes in chronic allograft rejection. Representative images of isogenic (left column) and allogenic (right column) kidney grafts 6 weeks after ktx are shown. Histological changes were evaluated on Masson Trichrome stains for fibrosis (MTC, 200-fold magnification, **A**,**B**), fibronectin staining for fibrosis (200-fold magnification, **C**,**D**) and CD3 stains for infiltrating T-lymphocytes (CD3, 200-fold magnification, **E**,**F**). Fibrosis is a hallmark of chronic rejection and is seen in MTC stain in blue and in fibronectin stain in red. MTC stain was accompanied by areas of inflammation (**B**, encircled) side-by-side with collagen deposition (**B**, arrows) after allogenic ktx. Fibronectin deposition was mainly localized in the renal cortex indicating focal scarring (**D**, encircled). In CD3 stain-dense tubulointerstitial infiltrates were seen in allografts mainly containing CD3 positive T-lymphocytes, visualized in red (**E**,**F**). Tubular autofluorescence in (**C**–**F**) appears green and was almost lost in allogenic kidney grafts indicating severe tubular destruction.

Chronic rejection in animals after allogenic ktx was characterized by histomorphological features of both, TCMR and ABMR (Figure 5). In allografts, dense infiltrates of CD3 positive T-cells were associated with antibody-mediated microvascular inflammation and peritubular capillaritis. In addition, in allogenic kidney grafts severe graft fibrosis as a hallmark of chronic rejection was present. In contrast, only mild inflammation and fibrosis was evident in isogenic kidney grafts without rejection. Consequently, semiquantitative analysis revealed significantly higher inflammation (3.1 ± 0.3 vs. 1.0 ± 0.5, *p* < 0.001) and fibrosis scores (43.8 ± 6.5% vs. 4.8 ± 1.2%, *p* < 0.001) in allografts compared to isografts (Table 3).

Of note, comparable to renal MRI, renal histology of allografts appeared heterogeneous with areas of severe fibrosis side-by-side with areas of tubular damage and inflammatory cell infiltration (Figure 5B).

## 4. Discussion

This study shows that MRI using mapping of T1 and T2 relaxation times as well as DWI allows the non-invasive characterization of chronic allograft rejection in mice. Evidence of inflammation and interstitial fibrosis in allografts with chronic rejection was associated with elevation of T1 and T2 relaxation times, reduction of CMD and increased heterogeneity of renal ADC values when compared to isogenic kidney grafts without rejection.

In our study we used a translational mouse model of chronic renal allograft rejection, described by Zarjou et al. [27], which is characterized by histomorphological features of both ABMR and TCMR. We adopted this model and found similar changes with interstitial inflammation, tubular atrophy, tubulointerstitial fibrosis, as well as endothelialitis and peritubular capillaritis after allogenic ktx [28]. For comparison, isogenic ktx of C57BL76-kidneys into C57BL76-mice was performed, which resulted in mild inflammation due to the ischemic injury, but no signs of rejection.

T1 and T2 mapping are promising for investigation of different renal pathologies [30]. Kidneys of healthy subjects show a distinguishable CMD on parameter maps of T1 relaxation time due to higher T1 values in the medulla compared with the cortex. This CMD is presumably caused by the higher free water content and higher mobility of water molecules in the medullary tubules and collecting ducts [31]. In our study, we could show that T1 relaxation time was significantly increased in allografts with chronic rejection compared to isografts at week 3 and 6 after transplantation in all anatomical layers. The physiological CMD was reduced after allogenic ktx as T1 increase was more pronounced in the cortex than in the ISOM. In isografts, a slight but not significant elevation of T1 relaxation time compared to control was observed and CMD was preserved. The elevation of T1 times in allografts in our study could be explained by interstitial inflammation, edema and fibrosis in the context of chronic renal allograft rejection. Furthermore, it has been shown that inhalation of oxygen results in decrease of renal T1 times [32,33]. As we could show in a previous study, chronic allograft rejection leads to severe allograft vasculopathy with endothelialitis, peritubular capillaritis and vascular occlusion [28]. These changes result in severe perfusion impairment and hypoxia of the kidney graft, which might also contribute to the prolongation of T1 relaxation time in allografts with chronic rejection. Our results are in line with previous studies showing that renal impairment is associated with increase of cortical T1 times and reduced CMD on T1 maps in various renal diseases such as chronic kidney disease [34], acute kidney injury or kidney transplantation [19]. In addition, a study of Friedlii et al. could show, by correlating MRI data and histology in patients after kidney transplantation, that reduced CMD of T1 values were associated with renal tubulointerstitial fibrosis in allografts [18].

T2 mapping showed only small and not significant differences between allografts with chronic rejection and isografts without rejection. In previous studies in mice with acute pathologies and severe inflammatory cell infiltration such as acute allograft rejection [16] and acute kidney injury [21], T2 times, particularly in the OSOM, were clearly elevated. In contrast, in a setting of chronic allograft rejection in the present study, acute inflammation and tissue edema were less pronounced, which explains only mild elevation of T2 times in this model. For comparison, in a multiparametric MRI study in patients who underwent ktx and healthy volunteers, Adams et al. found increased renal T2 times of the kidney graft, pronounced in the medulla and in the early period after surgery [35]. However, in contrast to other MRI parameters, results of T2 mapping were weak and no significant correlation with histopathology could be verified.

Intensive ongoing research in both animal models and humans pointed out the potential of DWI to identify different types of renal disease by reduced renal ADC values, with the most promising results in estimation of fibrosis in chronic kidney diseases and detection of acute inflammation in acute kidney injury and acute allograft rejection [36]. Contrary to our expectation, in the current study mean renal ADC values in mice with chronic allograft rejection and histological evidence of inflammation and fibrosis were not significantly different when compared to renal ADC in isografts without rejection. Nonetheless, visual assessment of ADC maps revealed clear differences between allografts with chronic rejection and isografts without rejection, i.e., parenchyma of allografts with chronic rejection appeared heterogeneous with areas of severely reduced ADC side-by-side with areas of increased ADC. In chronic rejection, pathophysiological mechanisms contributing to change in tissue diffusivity are complex, as different severities of cell infiltration occur together with cell swelling due to capillary leakage and edema, but also collagen deposition, necrosis and infarction. This may contribute to the observed heterogeneous renal pattern on ADC maps. For comparison, previous studies in humans have shown that a heterogeneous appearance of ADC maps is a qualitative sign associated with severe underlying histopathological renal changes after ktx [37,38]. In order to quantify this visual finding of tissue heterogeneity, histogram-based assessment of ADC maps was performed in our study. As a consequence of the heterogeneous texture of allografts, histogram-based analysis revealed a greater distribution of ADC values, resulting in a significant increase of heterogeneity parameters (SD, IQR and entropy) compared to isografts without rejection. ADC histogram analysis of the kidney has been reported to be particularly effective in reflecting the microstructure of renal tumors. Our findings show that histogram-based analysis may also be advantageous when characterizing chronic allograft pathology.

This study has limitations. First, the applied MRI-techniques (T1, T2 mapping and DWI) are not specific for distinct pathologies in renal allografts or for a certain histological feature. However, they allow multiparametric characterization of renal graft pathology, in addition to distribution and time course of parenchymal changes of the kidney graft, so that invasive procedures such as allograft biopsy may be guided. Second, our mouse model of chronic graft rejection shows severe changes in allografts under standardized conditions, so usability of T1, T2 mapping and DWI to detect allograft changes in milder degrees of rejection remains unclear. Third, to assess tissue heterogeneity diffusion tensor imaging (DTI) would have been an interesting additional application, as it allows information about three-dimensional diffusion and the microstructure of the renal graft. Last, we did not perform the direct correlation of MRI parameters with histological scores in each animal but analyzed renal histology in a subset of representative animals. This was considered reliable for this study, as the animal model is well characterized and variation of histological features between animals is expected to be low.

## 5. Conclusions

In conclusion, multiparametric MRI using mapping of T1 and T2 relaxation times as well as DWI allowed characterization of renal allograft pathology in a translational mouse model of chronic allograft rejection. In addition, these techniques have potential for non-invasive monitoring of long-term changes in renal allografts and might guide invasive assessment by allograft biopsy.

## Figures and Tables

**Table 1:** Functional MRI parameters after isogenic and allogenic kidney transplantation and in control animals.

	Control	Isogenic ktx		Allogenic ktx	
		w3	w6	w3	w6
T1 Cortex (ms)	1304 ± 10	1457 ± 52, ###	1393 ± 51 ###	1965 ± 53 ***	1899 ± 79 ***
T1 OSOM (ms)	1283 ± 11	1466 ± 56, ###	1399 ± 59 ###	2134 ± 61 ***	2036 ± 78 ***
T1 ISOM (ms)	1808 ± 19	1904 ± 45 ###	1859 ± 55	2282 ± 49 ***, §§§	2031 ± 66 **
T1 CMD (ms)	505 ± 20	447 ± 36	467 ± 55 ###	318 ± 30 **, §§	169 ± 53 ***
T2 Cortex (ms)	44 ± 1	48 ± 1	45 ± 2	48 ± 3	44 ± 3
T2 OSOM (ms)	42 ± 1	48 ± 1	44 ± 2	57 ± 7 ***	51 ± 4
T2 ISOM (ms)	54 ± 1	58 ± 2	55 ± 3	54 ± 8	50 ± 6
T2 CMD (ms)	10.5 ± 0.8	10 ± 2	9.6 ± 2.4	6.4 ± 5.7	5.5 ± 5.4
ADC Cortex (−10^−3^ mm^2^/s)	1.61 ± 0.06	1.29 ± 0.10	1.40 ± 0.11	1.47 ± 0.14	1.44 ± 0.17
ADC OM (−10^−3^ mm^2^/s)	1.72 ± 0.06	1.43 ± 0.10	1.56 ± 0.12	1.47 ± 0.06	1.27 ± 0.14 **

Mean values of different MRI parameters in the anatomic compartments of the grafts following isogenic and allogenic ktx as well as control kidneys are given as mean ± standard error of the mean (SEM). Significant differences compared to control kidneys are indicated with ** *p* < 0.01 and *** *p* < 0.001. Significant differences of allografts and isografts are indicated with ### *p* < 0.001. Temporal changes between week 3 (w3) and week 6 (w6) within one group are indicated with §§ *p* < 0.01 and §§§ *p* < 0.001. CMD, cortico-medullary difference (difference between ISOM and cortex); OM, outer medulla; OSOM, outer stripe of the outer medulla. Control *n* = 22, isogenic ktx *n* = 7, allogenic ktx *n* = 5.

**Table 2:** Histogram analysis of the ADC after isogenic and allogenic kidney transplantation.

	Isogenic ktx		Allogenic ktx	
	w3	w6	w3	w6
ADC_mean_ (×10^−3^ mm^2^/s)	1.39 ± 0.12	1.52 ± 0.10	1.16 ± 0.09	1.47 ± 0.14
SD (×10^−3^ mm^2^/s)	0.13 ± 0.02	0.15 ± 0.03	0.33 ± 0.02 ***	0.32 ± 0.03 **
Kurtosis	3.42 ± 0.30	2.96 ± 0.29	2.89 ± 0.23	3.31 ± 0.34
Skewness	−0.29 ± 0.29	0.14 ± 0.18	−0.14 ± 0.09	0.02 ± 0.19
Entropy	0.16 ± 0.008	0.17 ± 0.001	0.23 ± 0.004 ***	0.22 ± 0.007 *
IQR (×10^−3^ mm^2^/s)	0.17 ± 0.03	0.12 ± 0.03	0.45 ± 0.02 ***	0.43 ± 0.04 **

Significant differences between isogenic and allogenic kidney grafts at week 3 (w3) and week 6 (w6) after surgery are indicated with * *p* < 0.05, ** *p* < 0.01 and *** *p* < 0.001.

**Table 3:** Semiquantitative analysis of allogenic renal histopathology.

	Isogenic ktx	Allogenic ktx	*p*-Value
Fibrosis (%)	4.8 ± 1.2	43.8 ± 6.5	<0.001
Inflammation score	1.0 ± 0.5	3.1 ± 0.3	<0.001

## Data Availability

Data sharing not applicable.
